# Effect of Melatonin Supplementation on Reproductive Performance, Hormonal Profile and Antioxidant Status in Sheep Under Subtropical Conditions

**DOI:** 10.3390/ani16182954

**Published:** 2026-09-20

**Authors:** Chandan Singh, J. S. Rajoriya, A. K. Singh, B. K. Ojha, Deepak Ningwal, Madhuri Dhurvey, Atul Kumar Verma, S. S. Tomar, Amit Kumar Jha, S. S. Peepar, Mahak Singh, Megha Pande, Rohit Gupta

**Affiliations:** 1 Department of Veterinary Gynaecology and Obstetrics, College of Veterinary Science and Animal Husbandry, Rewa 486001, Madhya Pradesh, India; chandansngh347@gmail.com (C.S.); dr.brijeshojha@gmail.com (B.K.O.); deepak7nigwal@gmail.com (D.N.); drmadhuridhrv8@gmail.com (M.D.); 2 Department of Veterinary Physiology and Biochemistry, College of Veterinary Science and Animal Husbandry, Rewa 486001, Madhya Pradesh, India; dranil02@gmail.com; 3 Department of Veterinary Gynaecology and Obstetrics, College of Veterinary and Animal Science, Sardar Vallabhbhai Patel University of Agriculture & Technology, Modipuram, Meerut 250110, Uttar Pradesh, India; verma.atul1987@gmail.com; 4 Department of Animal Genetics and Breeding, Nanaji Deshmukh Veterinary Science University, Jabalpur 482004, Madhya Pradesh, India; dr_tomarss@rediffmail.com; 5 Department of Animal Genetics and Breeding, College of Veterinary Science and Animal Husbandry, Rewa 486001, Madhya Pradesh, India; jha.amit002@gmail.com; 6 ICAR-Indian Veterinary Research Institute, Bareilly 243122, Uttar Pradesh, India; shishantswaroop@gmail.com; 7 Animal Reproduction Laboratory, ICAR-Nagaland Centre, Medziphema 797106, Nagaland, India; mahaksinghivri@gmail.com; 8 Animal Physiology and Biochemistry Division, ICAR-Central Sheep and Wool Research Institute, Avikanagar 304501, Rajasthan, India; 9 ICAR-Central Institute for Research on Buffalo, Sub Campus, Nabha 147201, Patiala, India; rohit.gpt@icar.org.in

**Keywords:** melatonin, sheep, seasonal anestrus, reproductive performance, progesterone, estrogen, oxidative stress, antioxidant enzymes, subtropical climate

## Abstract

Limited information is available regarding the role of melatonin in regulating seasonal reproductive activity and oxidative stress in sheep under subtropical climatic conditions. Therefore, the present study evaluated the effect of melatonin administration on reproductive performance, hormonal profile, and antioxidant status in ewes during breeding and non-breeding seasons. A total of 48 clinically healthy multiparous ewes (2–5 years old) were equally divided into breeding and non-breeding season groups (*n* = 24 each). During each season, animals were allocated into four treatment groups: Group I (control; corn oil @ 1 mL/animal, s/c, *n* = 6), Group II (melatonin @ 10 mg/animal, s/c, *n* = 6), Group III (melatonin @ 20 mg/animal, s/c, *n* = 6), and Group IV (melatonin @ 40 mg/animal, s/c, *n* = 6). Melatonin administration significantly improved estrus response and conception rate during both seasons. During the breeding season, estrus response was observed in 33.33%, 100%, 100%, and 66.67% of animals in Groups I, II, III, and IV, respectively, whereas during the non-breeding season, estrus response was 0%, 83.33%, 100%, and 33.33%, respectively. Similarly, conception rates during the breeding season were 50%, 100%, 100%, and 66.67%, respectively, while corresponding values during the non-breeding season were 0%, 80%, 100%, and 50%. Serum estrogen concentration increased significantly (*p* < 0.05) in Groups II and III, particularly on day 7 during the breeding season and on day 14 during the non-breeding season. Progesterone concentration increased significantly (*p* < 0.05) on day 30 following 10 and 20 mg melatonin treatment during both seasons. Antioxidant enzyme activities, including glutathione peroxidase, superoxide dismutase, and catalase, were significantly enhanced following melatonin treatment. In conclusion, subcutaneous administration of melatonin improved reproductive performance, hormonal profile, and antioxidant status in sheep under subtropical conditions, with 20 mg melatonin/animal producing the most favorable responses, particularly during the non-breeding season.

## Simple Summary

Seasonal infertility is a major challenge in sheep production, particularly during the non-breeding season under subtropical climatic conditions. Melatonin, a hormone naturally produced by the pineal gland, is known to influence seasonal reproductive activity in sheep. The present study evaluated the effect of different doses of melatonin on estrus response, conception rate, reproductive hormones, and antioxidant status in ewes during both breeding and non-breeding seasons. Melatonin treatment, especially at a dose of 20 mg per animal, improved estrus induction and conception rate during the non-breeding season. Treated animals also showed improved progesterone and estrogen profiles along with increased antioxidant enzyme activities. These findings suggest that melatonin supplementation may help improve reproductive performance and reduce oxidative stress in sheep reared under subtropical conditions, particularly during periods of seasonal reproductive inactivity.

## 1. Introduction

Ewes are seasonally polyestrous animals, and their reproductive activity is characterized by alternating breeding and non-breeding seasons. The breeding season is marked by the regular occurrence of estrus and ovulation at intervals of approximately 16–17 days, whereas cyclic ovarian activity ceases during the non-breeding season [1]. Photoperiod, particularly changes in day length, is the primary environmental factor regulating seasonal reproduction in sheep. Maximum reproductive activity occurs during short-day periods, with the highest proportion of ewes exhibiting estrus and ovulatory activity during late summer, autumn, and early winter.

Under subtropical climatic conditions, sheep production is frequently constrained by reduced reproductive efficiency, particularly during the non-breeding season. Seasonal reproductive quiescence, primarily driven by photoperiod, results in delayed onset of estrus, reduced ovulation rates, impaired luteal function, and inconsistent fertility outcomes in sheep. These limitations pose a major challenge to achieving uniform lamb production and efficient reproductive management under subtropical production systems [2].

Melatonin, a neuroendocrine hormone secreted by the pineal gland, plays a crucial role in regulating seasonal reproduction in small ruminants by transducing photoperiodic information into endocrine signals that influence the hypothalamic–pituitary–gonadal axis. In sheep, melatonin modulates gonadotropin secretion, follicular development, ovulation, and corpus luteum function, thereby influencing estrus expression and fertility. Exogenous melatonin supplementation has been widely investigated as a management tool to advance the breeding season and improve reproductive performance under different production systems. Melatonin treatment has been shown to induce estrous cycles and increase ovulation and lambing rates in sheep [3]. Subcutaneous melatonin implants have also been successfully used to advance the breeding season and improve reproductive performance during anestrus in both highly seasonal and Mediterranean breeds of sheep [4,5]. Furthermore, melatonin implants mimic short-day photoperiodic responses without suppressing endogenous melatonin secretion [6]. Previous studies have also demonstrated that melatonin treatment improves fertility, prolificacy, and embryo viability in ewes [7,8]. In sheep and goats, exogenous melatonin administration during spring and summer prolongs circulating melatonin levels, resulting in enhanced gonadotropin secretion, earlier onset of the breeding season, and reduced prolactin secretion [9].

Apart from its photoperiodic role, melatonin is recognized as a potent antioxidant and cytoprotective molecule [10,11]. It directly scavenges reactive oxygen and nitrogen species and indirectly enhances endogenous antioxidant defense systems, including glutathione peroxidase, superoxide dismutase, and catalase [12]. Oxidative stress has been implicated in impaired ovarian steroidogenesis, reduced oocyte competence, compromised luteal activity, and poor uterine receptivity [13], all of which adversely affect reproductive performance in small ruminants.

Seasonal variations in reproductive hormones also contribute to fluctuations in fertility under subtropical conditions. Differences in progesterone and estrogen secretion between breeding and non-breeding seasons influence follicular dynamics, estrus expression, luteal maintenance, and early pregnancy establishment in sheep [1]. Several studies have reported that melatonin supplementation enhances luteal progesterone secretion and stabilizes estrogen profiles, thereby improving conception and lambing performance [7,8].

Although numerous studies have evaluated the effects of melatonin on reproductive traits in sheep, most investigations have focused either on fertility parameters or endocrine responses independently. Integrated studies simultaneously evaluating reproductive performance, hormonal profiles, and antioxidant status during both breeding and non-breeding seasons under subtropical conditions remain limited. Such integrated approaches are essential for understanding the multifaceted role of melatonin in reproductive regulation and for developing effective reproductive management strategies suitable for subtropical sheep production systems. Therefore, the present study was undertaken to evaluate the effect of melatonin supplementation on reproductive performance, endocrine profiles, and antioxidant status in sheep during the breeding and non-breeding seasons under subtropical climatic conditions.

## 2. Materials and Methods

### 2.1. Experimental Site and Climatic Conditions

The present study was conducted at the Department of Veterinary Gynaecology and Obstetrics, College of Veterinary Science and Animal Husbandry, Nanaji Deshmukh Veterinary Science University, Rewa, Madhya Pradesh, India (24.53° N, 81.30° E; approximately 304 m above mean sea level). The region experiences a humid subtropical climate with distinct seasonal variations in ambient temperature, relative humidity, and photoperiod. The average annual rainfall is approximately 1128 mm, with nearly 85% of the precipitation occurring during the southwest monsoon (June–September). During the breeding season (November–February), the mean ambient temperature ranged from 15 to 25 °C, relative humidity from 50 to 70%, and natural photoperiod from approximately 10.8 to 11.3 h/day, corresponding to a mean temperature–humidity index (THI) of approximately 60–68. During the non-breeding season (April–June), ambient temperatures ranged from 35 to 45 °C, relative humidity from 25 to 45%, and photoperiod from approximately 12.8 to 13.4 h/day, resulting in a mean THI of approximately 80–88.

### 2.2. Animals and Experimental Design

A total of 48 clinically healthy adult ewes, aged between 2 and 5 years and having lambed at least once, were included in the study. The animals were maintained under standard management and feeding conditions throughout the experimental period. All experimental procedures involving animals were reviewed and approved by the Institutional Animal Ethics Committee (IAEC), College of Veterinary Science and Animal Husbandry, Nanaji Deshmukh Veterinary Science University, Rewa, Madhya Pradesh, India (Approval No. 22/IAEC/Vety/Rewa/2020; approved on 30 December 2020). The study was conducted during both the breeding and non-breeding seasons, with 24 ewes assigned to each season. Within each season, the animals were randomly divided into four experimental groups (*n* = 6 per group) as follows: Group I (Control): corn oil administration only; Group II: melatonin administration at 10 mg/ewe; Group III: melatonin administration at 20 mg/ewe; Group IV: melatonin administration at 40 mg/ewe. The same experimental design and treatment protocol were followed during both breeding and non-breeding seasons.

### 2.3. Melatonin Preparation and Administration

Melatonin (Central Drug House [CDH] Pvt. Ltd., New Delhi, India) was dissolved in corn oil and administered as a single subcutaneous injection to the treatment groups. Stock solutions were prepared by dissolving melatonin powder in corn oil to obtain concentrations of 10 mg/mL, 20 mg/mL, and 40 mg/mL. Briefly, 100 mg, 200 mg, and 400 mg of melatonin were dissolved separately in 10 mL corn oil to prepare the respective concentrations. Ewes in Groups II, III, and IV received a single subcutaneous injection of melatonin corresponding to doses of 10 mg, 20 mg, and 40 mg per animal, respectively. Animals in the control group received corn oil alone without melatonin administration. The doses were selected to represent low, moderate, and high levels of melatonin supplementation in order to evaluate dose-dependent responses under field conditions, considering the previously reported beneficial effects of melatonin on reproductive performance in sheep [14,15].

### 2.4. Blood Collection

During both breeding and non-breeding seasons, blood samples were collected from all experimental animals by aseptic jugular venipuncture using sterile 18-gauge needles. Sampling was performed on day 0 (day of melatonin administration) and subsequently on days 7, 14, and 30 post-treatment for the estimation of estrogen, progesterone, and antioxidant enzyme activities. Collected blood samples were allowed to clot at room temperature, followed by serum separation by centrifugation at 3000 rpm for 10 min. The separated serum was aliquoted and stored at −20 °C until further hormonal and biochemical analyses.

### 2.5. Estrus Detection

Estrus detection was carried out through visual observation of behavioral signs along with examination of cervico-vaginal discharge in the presence of teaser rams. Behavioral signs considered indicative of estrus included restlessness, frequent bleating, tail wagging, vulvar swelling, and acceptance of mounting by the male. Ewes that stood firmly to be mounted by teaser rams were considered to be in estrus. Ewes detected in estrus were naturally mated with healthy, proven fertile rams maintained under identical management conditions. The same breeding protocol was followed for all treatment groups within each season to minimize variation associated with mating management and ram fertility.

### 2.6. Pregnancy Diagnosis

**Figure 1:**
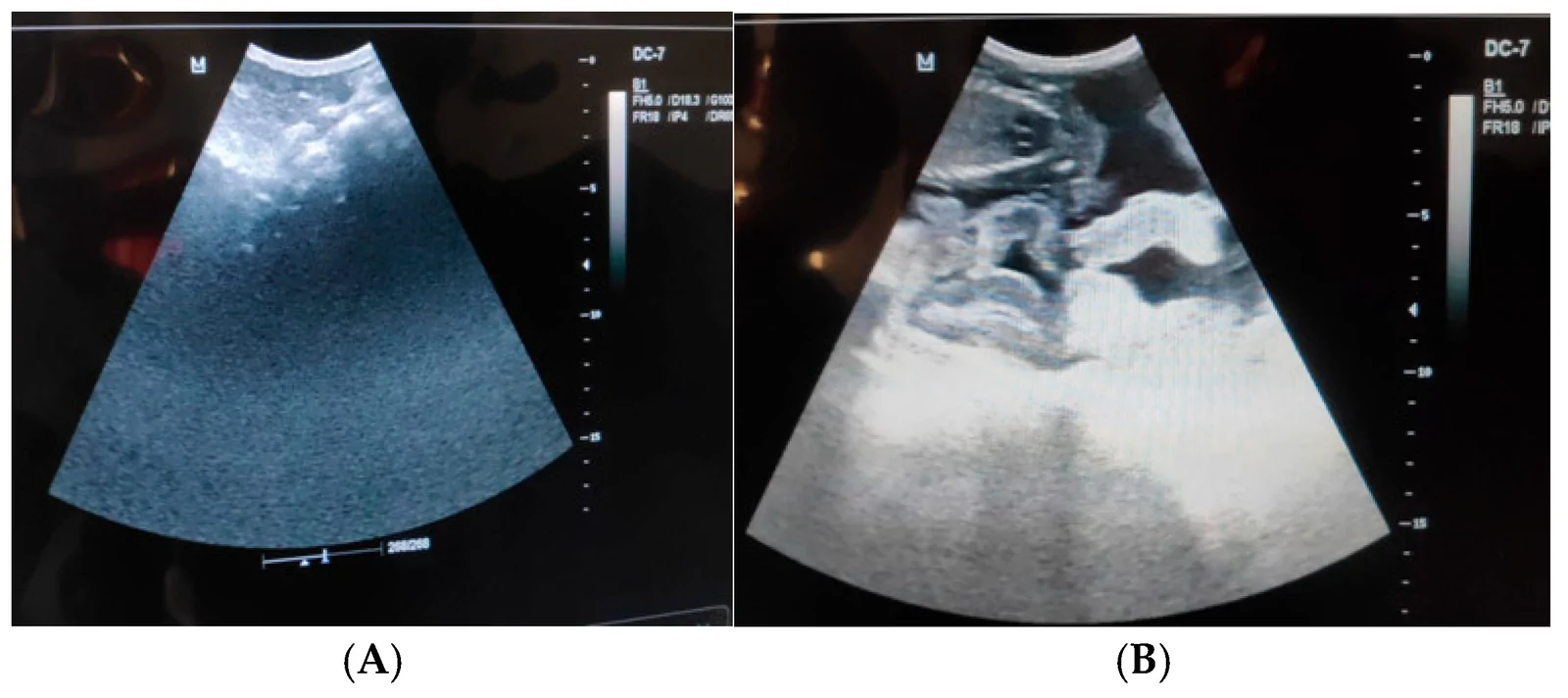
Representative trans-abdominal ultrasonographic images recorded on day 28 post-breeding in ewes using a 7.5 MHz convex probe. (**A**) Early embryonic vesicle observed within the uterine lumen. (**B**) Visualization of fetal structures confirming pregnancy.

Pregnancy diagnosis was performed on day 28 post-breeding by trans-abdominal ultrasonographic examination using a real-time B-mode ultrasonography unit equipped with a 7.5 MHz convex probe (Mindray, Mahwah, NJ, USA). Detection of embryonic vesicles and fetal structures (Figure 1) was considered confirmation of pregnancy. Subsequently, all pregnant ewes were monitored until parturition, and lambing outcomes were recorded.

### 2.7. Estrogen and Progesterone Assays

Serum estrogen and progesterone concentrations were estimated on days 0, 7, 14, and 30 following treatment. Serum estrogen concentration was quantified using a commercially available ELISA kit (ELISA kit (G-Biosciences, St. Louis, MO, USA)), whereas serum progesterone concentration was quantified using a commercially available ELISA kit (Cayman Chemical, Ann Arbor, MI, USA), according to the manufacturers’ instructions. The estrogen assay had a sensitivity of 10 pg/mL, with intra-assay and inter-assay coefficients of variation of <8% and <10%, respectively. The progesterone assay had an intra-assay coefficient of variation of <10% and an inter-assay coefficient of variation of <12%, as specified by the manufacturer. All serum samples were analyzed in duplicate, and calibration curves (R^2^ > 0.98) were verified for each assay batch to ensure analytical accuracy and reproducibility.

### 2.8. Glutathione Peroxidase (GPx) Assay

Serum glutathione peroxidase (GPx) activity was determined using a commercially available Glutathione Peroxidase Assay Kit (Cayman Chemical, Ann Arbor, MI, USA) following the manufacturer’s protocol. Glutathione peroxidase is an important antioxidant enzyme that catalyzes the reduction of hydroperoxides, including hydrogen peroxide, using reduced glutathione as an electron donor, thereby protecting cells against oxidative damage.

### 2.9. Superoxide Dismutase (SOD) Assay

Serum superoxide dismutase (SOD) activity was determined following the method of Madesh and Balasubramanian [16], with slight modifications. Briefly, the reaction mixture (300 μL total volume per well) consisted of 120 μL phosphate-buffered saline (PBS), 10 μL serum sample, 5 μL of 1.25 mM MTT solution, and 15 μL freshly prepared 1 mM pyrogallol solution. For blank preparation, the serum sample was replaced with PBS. After incubation for 15 min, 15 μL dimethyl sulfoxide (DMSO) was added, and absorbance was measured at 570 nm using a microplate reader. SOD activity was expressed as units/mL.

### 2.10. Catalase Assay

Serum catalase activity was estimated following the method described by Korolyuk et al. [17], with slight modifications. Briefly, 0.1 mL serum sample was incubated with 2.0 mL substrate solution containing hydrogen peroxide (10 μmol/mL H_2_O_2_) prepared in sodium–potassium phosphate buffer (pH 7.4) for 10 min. The reaction was terminated by adding 1.0 mL ammonium molybdate solution (32.4 mmol/L), and the absorbance of the resulting yellow-colored complex was measured at 410 nm using a spectrophotometer. Catalase activity was expressed as kU/L.

### 2.11. Statistical Analysis

All statistical analyses were performed using IBM SPSS Statistics software (Version 27.0; IBM Corp., Armonk, NY, USA). Data were initially tested for normality using the Shapiro–Wilk test. Results are presented as mean ± standard error of the mean (SEM). Reproductive parameters, including estrus response, conception rate, and lambing rate, were analyzed using the generalized linear model (GLM) procedure with binomial distribution and logit link function, considering treatment group (control, 10, 20, and 40 mg melatonin) as the fixed effect. Hormonal parameters (estrogen and progesterone) and antioxidant enzyme activities [glutathione peroxidase (GPx), superoxide dismutase (SOD), and catalase (CAT)] were analyzed by repeated-measures ANOVA, with treatment group and sampling day (0, 7, 14, and 30) included in the model. When significant effects or treatment × sampling day interactions were detected, mean differences were compared using Tukey’s post hoc test. Differences were considered statistically significant at *p* < 0.05.

## 3. Results

### 3.1. Reproductive Performance

Melatonin treatment significantly (*p* < 0.05) affected reproductive performance during both breeding and non-breeding seasons (Table 1). During the breeding season, estrus response differed significantly (*p* < 0.05) among treatment groups. Groups II and III exhibited 100% estrus response, whereas Group I showed the lowest response (33.33%). Conception and lambing rates were also significantly (*p* < 0.05) higher in Groups II and III compared with Groups I and IV.

During the non-breeding season, estrus response differed significantly (*p* < 0.01) among groups. Group III showed the highest estrus response (100%), followed by Group II (83.33%), whereas no estrus was observed in the control group. Conception and lambing rates were significantly (*p* < 0.05) higher in Group III compared with the remaining groups. Overall, melatonin administration at 20 mg/ewe resulted in the best reproductive performance during both breeding and non-breeding seasons.

### 3.2. Serum Estrogen Concentration

Serum estrogen concentration varied significantly (*p* < 0.05) among treatment groups and sampling days during both breeding and non-breeding seasons (Table 2). During the breeding season, estrogen concentration increased significantly (*p* < 0.05) on day 7 in Groups II and III compared with Groups I and IV, with Group III recording the highest value. Group I exhibited peak estrogen concentration on day 14, whereas Group IV showed a gradual increase, reaching the highest concentration on day 30.

During the non-breeding season, Group II showed significantly (*p* < 0.05) higher estrogen concentration on day 7 compared with Groups I and IV. A marked increase in estrogen concentration was observed in Group III on day 14, which was significantly (*p* < 0.05) higher than all other groups. Estrogen concentration declined by day 30 in all groups. Overall, melatonin administration at 20 mg/ewe resulted in the greatest increase in serum estrogen concentration during both seasons.

### 3.3. Serum Progesterone Concentration

Serum progesterone concentration differed significantly (*p* < 0.05) among treatment groups and sampling days during both breeding and non-breeding seasons (Table 3). During the breeding season, progesterone concentration decreased on day 7 in Groups II and III, followed by a marked increase on days 14 and 30. Group III recorded the highest progesterone concentration on day 30, followed by Group II, whereas Groups I and IV showed comparatively lower concentrations throughout the study period.

During the non-breeding season, progesterone concentration remained relatively stable up to day 14 in most groups; however, a pronounced increase was observed on day 30 in Groups II and III. Group III exhibited the highest progesterone concentration among all groups. In contrast, Groups I and IV showed comparatively moderate changes during the experimental period. These findings indicate enhanced luteal activity and progesterone secretion following melatonin treatment, particularly at 20 mg/ewe.

### 3.4. Serum Glutathione Peroxidase Activity

Serum glutathione peroxidase (GPx) activity differed significantly (*p* < 0.05) among treatment groups and sampling days during both breeding and non-breeding seasons (Table 4). During the breeding season, GPx activity increased significantly (*p* < 0.05) from day 7 onward in Groups II, III, and IV compared with the control group, with peak activity observed on day 14, particularly in Groups III and IV. Although GPx activity declined slightly by day 30, values remained significantly higher (*p* < 0.05) than baseline levels in melatonin-treated groups.

A similar trend was observed during the non-breeding season, where Groups II, III, and IV showed significantly higher (*p* < 0.05) GPx activity than the control group from day 7 onward. Peak GPx activity was observed on day 14 in Groups III and IV, followed by a moderate decline at day 30. Minimal temporal variation was observed in the control group during both seasons.

### 3.5. Serum Superoxide Dismutase Activity

Serum superoxide dismutase (SOD) activity varied significantly (*p* < 0.05) among treatment groups and sampling days during both breeding and non-breeding seasons (Table 5). During the breeding season, SOD activity increased significantly (*p* < 0.05) in Groups II, III, and IV from day 7 onward compared with Group I. Group IV showed the highest SOD activity on day 7, whereas Group III recorded the highest activity on day 14. Although SOD activity declined slightly by day 30, treated groups maintained significantly higher values than the control group.

During the non-breeding season, SOD activity did not differ significantly among groups at day 0. However, from day 7 onward, Groups II, III, and IV showed significantly higher SOD activity than Group I. Groups III and IV exhibited the highest SOD activity on day 14, followed by a decline on day 30. In contrast, the control group showed minimal variation throughout the study period.

### 3.6. Serum Catalase Activity

Serum catalase activity differed significantly (*p* < 0.05) among treatment groups and sampling days during both breeding and non-breeding seasons (Table 6). During the breeding season, catalase activity increased significantly (*p* < 0.05) in Groups II, III, and IV from day 7 onward compared with the control group. Peak catalase activity was observed on day 14 in Groups III and IV, with Group IV showing the highest value. Catalase activity remained significantly elevated in melatonin-treated groups on day 30 compared with the control group.

Similarly, during the non-breeding season, catalase activity was significantly (*p* < 0.05) higher in Groups II, III, and IV than in the control group from day 7 onward. Groups III and IV showed the highest catalase activity on day 14, followed by a moderate decline at day 30. Minimal temporal variation was observed in the control group during both seasons, whereas melatonin-treated groups exhibited significant (*p* < 0.05) increases in catalase activity over time.

## 4. Discussion

The present study demonstrated that melatonin administration significantly improved reproductive performance, endocrine profile, and antioxidant status in ewes during both breeding and non-breeding seasons under subtropical climatic conditions. Among the different treatment groups, melatonin administration at 20 mg/ewe produced the most consistent improvement in estrus response, conception rate, lambing rate, ovarian hormone secretion, and antioxidant enzyme activity. These findings suggest that melatonin effectively modulated seasonal reproductive activity and oxidative balance in sheep maintained under subtropical environmental conditions.

During the breeding season, estrus induction was observed in 50–66.66% of ewes across treatment groups, whereas ewes treated with 10 mg and 20 mg melatonin exhibited 100% estrus response. The comparatively limited stimulatory effect of melatonin during this period may be attributed to naturally elevated endogenous melatonin secretion under short-day photoperiodic conditions, which already sustains hypothalamic–pituitary–gonadal axis activity and reproductive cyclicity in sheep. Similar observations have been reported previously, indicating that melatonin supplementation during the physiological breeding season produces only marginal improvement in estrus expression and fertility in short-day breeders [13,18]. In contrast, the comparatively lower estrus response observed in ewes receiving 40 mg melatonin suggests a possible dose-dependent inhibitory or desensitizing effect associated with excessive melatonin exposure. Supra-physiological melatonin concentrations may alter GnRH pulsatility and ovarian responsiveness through neuroendocrine feedback mechanisms, thereby affecting normal reproductive signaling pathways [11,19].

During the non-breeding season, control ewes failed to exhibit estrus activity, confirming deep seasonal anestrus under long-day photoperiodic conditions. Melatonin administration effectively induced estrus in ewes treated with 10 mg and 20 mg melatonin, whereas a comparatively reduced response was again observed in the 40 mg group. These findings indicated that melatonin, when administered at an optimal dose, may mimic short-day photoperiodic signals and reactivates reproductive neuroendocrine pathways in sheep. Similar observations have been reported previously, where melatonin treatment restored ovarian cyclicity, enhanced estrus response, and improved reproductive efficiency during seasonal anestrus in sheep [13,18]. Recent studies have further demonstrated that melatonin supplementation enhances follicular steroidogenesis, luteal activity, and antioxidant defense mechanisms under environmentally stressful conditions, thereby improving reproductive performance in ewes [20,21].

The high estrus response achieved following a single subcutaneous administration in the present study highlights the practical utility of melatonin-based protocols for field-level management of seasonal reproductive quiescence under subtropical production systems. Occasional estrus-like behavior observed in control ewes may be associated with the ram effect and social stimulation; however, the absence of sustained estrus expression confirms that endocrine intervention is essential for overcoming seasonal reproductive suppression associated with long-day photoperiods. Moreover, melatonin-based protocols have been reported to alleviate reproductive quiescence, advance the onset of the breeding season, and improve estrus synchronization and fertility in ewes bred during seasonal anestrus, thereby facilitating more synchronized breeding and lambing management [19,22,23,24,25].

The hormonal profile observed in the present study further supports the stimulatory role of melatonin on ovarian activity and follicular steroidogenesis. During the breeding season, serum estrogen concentration increased transiently following melatonin administration, particularly in ewes treated with 10 mg and 20 mg melatonin. The highest estrogen concentration was recorded on day 7 post-treatment in the 20 mg group, indicating enhanced follicular recruitment and increased ovarian steroidogenic activity following melatonin supplementation. Melatonin is known to modulate reproductive function through its actions on the hypothalamic–pituitary–gonadal axis as well as through direct effects on granulosa and luteal cells, where it influences follicular development, aromatase expression, and estradiol biosynthesis [26]. Experimental studies in granulosa–lutein cells have demonstrated that melatonin stimulates aromatase expression and estradiol production through melatonin receptor-mediated signaling pathways, thereby enhancing follicular endocrine function and oocyte competence [27]. Similar stimulatory effects of melatonin on follicular growth, meiotic resumption, mitochondrial activity, and oocyte developmental competence have also been reported in sheep oocytes cultured in vitro [21].

The comparatively lower or delayed estrogen response observed in the high-dose melatonin group in the present study suggests that excessive melatonin exposure may alter normal steroidogenic regulation in a dose-dependent manner. Although physiological melatonin concentrations generally support ovarian function, supra-physiological levels may disrupt endocrine homeostasis and modify ovarian responsiveness through altered receptor sensitivity or neuroendocrine feedback mechanisms. Previous reports have indicated that melatonin exerts complex regulatory effects on reproductive hormones depending on dose, duration of exposure, and physiological status of the animal [26,28]. Collectively, these findings support the view that moderate melatonin supplementation enhances ovarian steroidogenesis and reproductive endocrine activity.

During the non-breeding season, control ewes maintained comparatively low serum estrogen concentrations throughout the study period, reflecting ovarian inactivity associated with seasonal anestrus and photoperiod-induced suppression of hypothalamic–pituitary–ovarian activity. In contrast, melatonin-treated ewes, particularly those receiving 20 mg melatonin, exhibited a marked increase in estrogen concentration on day 14 post-treatment, suggesting enhanced follicular activity and increased estrogen secretion following melatonin administration. The delayed estrogen peak observed during the non-breeding season, compared with the breeding season, may reflect the time required for exogenous melatonin to overcome long-day photoperiodic inhibition and re-establish normal reproductive neuroendocrine signaling. Melatonin is known to regulate reproductive cyclicity through modulation of GnRH secretion and ovarian responsiveness, thereby promoting follicular development and estradiol synthesis in seasonal breeders [13,18]. Similar stimulatory effects of melatonin on follicular steroidogenesis and estrogen production have also been reported in granulosa cells and developing follicles across several mammalian species [26,27]. The significantly elevated estrogen concentration observed during estrus in the present study is consistent with the normal pre-ovulatory rise in estradiol reported previously in small ruminants and reflects enhanced follicular maturation and ovulatory readiness following melatonin administration [15,19].

Similarly, serum progesterone concentration increased significantly following melatonin administration during both breeding and non-breeding seasons. In the present study, the highest progesterone concentration was consistently observed in ewes treated with 20 mg melatonin, indicating enhanced corpus luteum function and improved luteal activity at this dose. The comparatively greater progesterone response observed in melatonin-treated ewes may reflect improved ovulatory response, enhanced luteal development, and better maintenance of early pregnancy following melatonin supplementation. Progesterone plays a critical role in uterine receptivity, embryo implantation, and maintenance of gestation; therefore, the elevated progesterone concentrations observed in the present study may have contributed directly to the improved conception and lambing rates recorded in melatonin-treated groups.

The present findings are consistent with previous reports demonstrating that melatonin supplementation enhances ovarian endocrine activity and reproductive performance in sheep. Song et al. [15] reported significantly higher estrogen concentration, pregnancy rate, embryo yield, and lambing performance in melatonin-treated ewes subjected to superovulation and embryo transfer protocols. Similarly, Duan et al. [14] demonstrated that melatonin implantation during seasonal anestrus increased luteal diameter, progesterone secretion, and steroidogenic activity of granulosa and luteal cells, thereby improving pregnancy establishment in ewes. Recent evidence further suggests that melatonin improves early pregnancy establishment by protecting trophoblast cells against oxidative and hypoxic stress. Viola et al. [28] demonstrated that melatonin reduced apoptosis, modulated autophagy, improved trophoblast cell functionality, and enhanced cellular resilience under hypoxic conditions in ovine trophoblast cells, thereby supporting conceptus survival during early gestation. Such cytoprotective and antioxidant effects may have contributed to the improved conception and lambing rates observed in the present study.

The comparatively lower progesterone response observed in the high-dose melatonin group in the present study suggests that excessive melatonin exposure may not proportionally enhance luteal activity and could potentially disrupt endocrine homeostasis through altered receptor sensitivity, impaired steroidogenic regulation, or modified neuroendocrine feedback mechanisms. Although melatonin exerts beneficial reproductive and antioxidant effects at physiological or moderate concentrations, several studies have demonstrated that its actions are highly dose-dependent. Adriaens et al. [29] reported that high melatonin concentrations adversely affected folliculogenesis, oocyte maturation, and steroidogenesis, while only lower concentrations exerted protective and beneficial effects on ovarian function. Similarly, studies on ovine embryos have shown that elevated melatonin concentrations during post-warming culture increased oxidative and apoptotic indices and reduced embryo viability, whereas lower concentrations improved embryo development and cellular function [30]. These findings indicate that excessive melatonin supplementation may exert paradoxical or inhibitory effects on reproductive physiology, emphasizing the importance of optimizing melatonin dosage to achieve favorable endocrine and reproductive outcomes in sheep.

The present study demonstrated a marked improvement in conception rate following melatonin administration during both breeding and non-breeding seasons. During the breeding season, conception rate reached 100% in ewes treated with 10 mg and 20 mg melatonin, whereas comparatively lower conception rates were observed in the control and high-dose groups. The improved conception response at moderate melatonin doses may be attributed to enhanced estrus synchronization, improved follicular maturation, optimized luteal function, and a more favorable uterine environment for fertilization and early embryo survival. Melatonin is known to positively influence ovarian steroidogenesis, oocyte competence, embryo quality, and maternal recognition of pregnancy through its endocrine and antioxidant actions [19,27].

During the non-breeding season, control ewes failed to conceive, confirming severe reproductive suppression associated with seasonal anestrus under long-day photoperiodic conditions. In contrast, melatonin-treated ewes exhibited markedly improved conception rates, with the highest response again observed in the 20 mg group. These findings indicate that melatonin improved reproductive performance during seasonal anestrus, possibly by mimicking short-day photoperiodic signals and enhancing endocrine and antioxidant responses. Similar improvements in conception rate, pregnancy establishment, and lambing performance following melatonin administration have been reported previously in sheep bred out of season and in estrus-synchronized ewes [14,23,25].

Recent studies further suggest that melatonin improves reproductive success by enhancing embryo developmental competence, reducing oxidative stress, and protecting trophoblast and embryonic cells from apoptosis and hypoxic injury [22,28]. Improved antioxidant defense and maintenance of luteal progesterone secretion may therefore have contributed to the higher conception and lambing rates observed in melatonin-treated ewes in the present study. However, the comparatively reduced conception response observed in the high-dose melatonin group again indicates that excessive melatonin exposure may adversely affect reproductive endocrine balance, emphasizing the importance of dose optimization for achieving maximal reproductive efficiency under subtropical production conditions.

The present study further demonstrated that melatonin administration significantly enhanced antioxidant defense mechanisms in ewes, as evidenced by increased activities of superoxide dismutase (SOD), glutathione peroxidase (GPx), and catalase during both breeding and non-breeding seasons. The antioxidant response was dose-, time-, and season-dependent, with peak enzyme activities generally observed between days 7 and 14 post-treatment, particularly in ewes receiving moderate melatonin doses. These findings support the well-established role of melatonin as both a direct free-radical scavenger and an indirect regulator of endogenous antioxidant systems. Melatonin enhances cellular redox balance, protects sulfhydryl groups from oxidative damage, stabilizes mitochondrial function, and stimulates antioxidant enzyme activity, thereby improving resistance against oxidative stress [31,32,33,34].

The coordinated increase in SOD, GPx, and catalase activities observed in the present study reflects the complementary and interdependent functions of these antioxidant enzymes. Superoxide dismutase catalyzes the conversion of superoxide radicals into hydrogen peroxide, which is subsequently detoxified by catalase and GPx into non-toxic end products. The simultaneous elevation of SOD and catalase activities following melatonin treatment therefore indicates activation of an integrated antioxidant defense cascade. Similar improvements in antioxidant enzyme activity following melatonin supplementation have been reported previously in livestock and reproductive tissues subjected to oxidative stress [35,36]. Melatonin additionally contributes to antioxidant protection through its metabolites, including AFMK and AMK, which possess independent free-radical scavenging properties and further amplify the antioxidant cascade [37,38].

Seasonal comparison revealed comparatively higher antioxidant enzyme activities during the breeding season than during the non-breeding season, indicating a better basal antioxidant status under thermoneutral short-day conditions. Reduced antioxidant enzyme activities during the non-breeding season may be associated with increased oxidative stress, elevated ambient temperature, lipid peroxidation, and enhanced utilization of antioxidant reserves under heat-stress conditions. Heat stress is well recognized to impair reproductive efficiency through oxidative damage to ovarian tissues, oocytes, embryos, and placental cells, ultimately affecting estrus expression, fertilization, embryonic survival and fetal development in sheep [39,40,41]. Importantly, melatonin administration during the non-breeding season significantly restored antioxidant enzyme activities in the present study, indicating its protective role against thermal and oxidative stress associated with seasonal reproductive quiescence.

Recent evidence further suggests that melatonin-mediated antioxidant protection extends beyond systemic oxidative balance to the embryo–maternal interface and early embryonic development. Viola et al. [28,42] demonstrated that exogenous melatonin improves embryo–maternal cross-talk, enhances placental and endometrial remodeling, reduces trophoblast apoptosis, and promotes cellular resilience under hypoxic conditions in sheep. Similarly, melatonin supplementation has been shown to improve embryo viability, developmental competence, mitochondrial activity, and expression of oxidative stress response genes in fresh and vitrified embryos by reducing reactive oxygen species and apoptotic damage [5,22,43]. These findings collectively indicate that melatonin not only enhances antioxidant defense mechanisms but also protects reproductive tissues and developing embryos against oxidative and environmental stressors, thereby contributing to improved conception and lambing outcomes. Overall, the findings of the present study demonstrate that melatonin acts as an effective endocrine and antioxidant modulator capable of improving reproductive efficiency and oxidative status in sheep under subtropical climatic conditions. Among the tested doses, administration of 20 mg melatonin/ewe produced the most favorable reproductive and biochemical responses during both breeding and non-breeding seasons, whereas higher doses were comparatively less effective, indicating a dose-dependent physiological response. These results suggest that melatonin supplementation may serve as a practical strategy for enhancing fertility and alleviating seasonal reproductive limitations in sheep reared under subtropical environments.

Despite these encouraging findings, there were certain limitations in the present study. The experiment was conducted using a relatively small number of animals under a single management system and subtropical environment, which may limit the wider applicability of the results. In addition, ovarian ultrasonography and gonadotropin profiling were not performed. Nevertheless, the consistent improvements observed in reproductive performance, hormonal profile, and antioxidant enzyme activities provide strong evidence for the beneficial effects of melatonin supplementation in ewes. Future studies involving larger populations, multiple breeds, different production systems, and more comprehensive endocrine and oxidative stress assessments will help validate and extend the present findings.

## 5. Conclusions

Seasonal reproductive inactivity and oxidative stress considerably impair reproductive performance in sheep under subtropical climatic conditions, particularly during the non-breeding season. The present study concluded that melatonin administration improved estrus response, conception rate, hormonal profile, and antioxidant status in ewes during both breeding and non-breeding seasons. Among the evaluated doses, 20 mg melatonin/ewe produced the most favorable reproductive and biochemical responses. Melatonin supplementation was associated with increased estrogen and progesterone concentrations together with enhanced antioxidant enzyme activities, including GPx, SOD, and catalase. Therefore, melatonin administration may be considered an effective reproductive management strategy for improving fertility and reducing seasonal reproductive constraints in sheep under the subtropical production and edaphoclimatic conditions of the present study. However, the study was limited by the relatively small sample size and evaluation under a single management and environmental system; therefore, further large-scale studies are needed to validate its long-term field applicability across different breeds and climatic regions.

## Figures and Tables

**Table 1:** Effect of melatonin treatment on reproductive performance in ewes during breeding and non-breeding seasons.

Season	Group	No. of Animals	Estrus Response (%)	Naturally Mated (*n*)	Conception Rate (%)	Lambing Rate (%)
Breeding	I	6	33.33 ^c^ (2)	2	50 ^b^ (1)	50 ^b^ (1)
	II	6	100 ^a^ (6)	6	100 ^a^ (6)	100 ^a^ (6)
	III	6	100 ^a^ (6)	6	100 ^a^ (6)	100 ^a^ (6)
	IV	6	66.66 ^b^ (4)	4	66.66 ^b^ (4)	66.66 ^b^ (4)
Non-breeding	I	6	0 ^c^ (0)	0	0 ^c^ (0)	0 ^c^ (0)
	II	6	83.33 ^b^ (5)	5	80 ^b^ (4)	80 ^b^ (4)
	III	6	100 ^a^ (6)	6	100 ^a^ (6)	100 ^a^ (6)
	IV	6	33.33 ^c^ (2)	2	50 ^bc^ (1)	50 ^bc^ (1)

Different superscripts within columns indicate significant differences (*p* < 0.05).

**Table 2:** Serum estrogen concentration (pg/mL) in ewes during breeding and non-breeding seasons following melatonin treatment (Mean ± SEM).

Season	Group	Day 0	Day 7	Day 14	Day 30
Breeding	I	2.61 ± 0.12 ^A^	3.91 ± 1.01 ^ABa^	6.41 ± 1.29 ^Bb^	3.21 ± 0.15 ^ABa^
	II	2.78 ± 0.23 ^A^	8.15 ± 1.28 ^Bb^	2.85 ± 0.15 ^Aa^	3.53 ± 0.11 ^Aa^
	III	2.52 ± 0.15 ^A^	11.43 ± 0.43 ^Bb^	3.01 ± 0.09 ^Aab^	3.44 ± 0.16 ^Aa^
	IV	2.39 ± 0.17 ^A^	3.29 ± 1.13 ^a^	3.92 ± 1.12 ^ab^	5.66 ± 0.16 ^b^
Non-breeding	I	2.76 ± 0.25	2.54 ± 0.22 ^ab^	3.98 ± 0.99 ^a^	2.62 ± 0.27
	II	2.09 ± 0.19	5.71 ± 1.72 ^b^	4.94 ± 1.47 ^a^	2.63 ± 0.21
	III	2.91 ± 0.16 ^A^	2.56 ± 0.17 ^Aab^	9.99 ± 1.12 ^Bb^	3.05 ± 0.21 ^A^
	IV	2.41 ± 0.21	2.23 ± 0.24 ^a^	4.97 ± 1.59 ^a^	2.34 ± 0.15

Within each season, different uppercase superscripts (A–B) within the same row indicate significant differences among sampling days for the same treatment group. Different lowercase superscripts (a–b) within the same column indicate significant differences among treatment groups on the same sampling day (*p* < 0.05). Cells without superscripts indicate that no significant differences were detected (*p* > 0.05).

**Table 3:** Serum progesterone concentration (ng/mL) in ewes during breeding and non-breeding seasons following melatonin treatment (Mean ± SEM).

Season	Group	Day 0	Day 7	Day 14	Day 30
Breeding	I	2.61 ± 0.43 ^A^	2.50 ± 0.89 ^Ab^	1.97 ± 0.87 ^Aa^	5.35 ± 0.21 ^Ba^
	II	2.67 ± 0.14 ^B^	0.74 ± 0.11 ^Aa^	8.65 ± 0.13 ^Cb^	10.13 ± 0.17 ^Cb^
	III	2.92 ± 0.23 ^B^	0.59 ± 0.03 ^Aa^	9.14 ± 0.23 ^Cb^	11.59 ± 0.33 ^Db^
	IV	2.59 ± 0.21 ^A^	1.92 ± 0.36 ^Aab^	2.01 ± 0.89 ^Aa^	5.66 ± 1.08 ^Ba^
Non-breeding	I	1.63 ± 0.27	2.37 ± 0.68	1.97 ± 0.33 ^b^	3.48 ± 1.21 ^a^
	II	2.14 ± 0.69 ^Ab^	2.22 ± 1.03 ^A^	1.89 ± 1.15 ^Ab^	7.91 ± 1.19 ^Bb^
	III	1.97 ± 0.11 ^Ba^	2.68 ± 0.15 ^B^	0.69 ± 0.03 ^Aa^	9.12 ± 0.36 ^Cb^
	IV	1.85 ± 0.09	3.03 ± 0.93 ^a^	1.67 ± 0.43 ^b^	4.13 ± 1.54 ^a^

Within each season, different uppercase superscripts (A–D) within the same row indicate significant differences among sampling days for the same treatment group. Different lowercase superscripts (a–b) within the same column indicate significant differences among treatment groups on the same sampling day (*p* < 0.05). Cells without superscripts indicate that no significant differences were detected (*p* > 0.05).

**Table 4:** Serum glutathione peroxidase (GPx) activity (nmol/min/mL) in ewes during breeding and non-breeding seasons following melatonin treatment (Mean ± SEM).

Season	Group	Day 0	Day 7	Day 14	Day 30
Breeding	I	70.09 ± 1.01	74.24 ± 1.59 ^a^	70.98 ± 1.46 ^a^	69.36 ± 1.35 ^a^
	II	70.41 ± 0.76 ^A^	81.43 ± 1.47 ^Cb^	75.64 ± 1.38 ^Ba^	75.03 ± 1.12 ^ABab^
	III	72.09 ± 1.17 ^A^	83.59 ± 0.99 ^Cbc^	89.39 ± 1.19 ^Db^	76.26 ± 0.78 ^Bb^
	IV	70.47 ± 0.89 ^A^	87.27 ± 1.62 ^BCc^	91.63 ± 0.86 ^Cb^	82.33 ± 2.24 ^Bc^
Non-breeding	I	59.47 ± 0.96	59.68 ± 1.33 ^a^	59.04 ± 0.66 ^a^	58.39 ± 0.69
	II	56.96 ± 0.92 ^A^	60.85 ± 0.66 ^Ba^	62.46 ± 0.93 ^Ba^	59.47 ± 0.78 ^AB^
	III	58.06 ± 1.16 ^A^	70.96 ± 0.81 ^Bb^	75.66 ± 1.01 ^Bb^	61.22 ± 1.67 ^A^
	IV	58.04 ± 1.78 ^A^	68.54 ± 2.37 ^BCb^	73.21 ± 2.72 ^Cb^	62.46 ± 2.44 ^AB^

Within each season, different uppercase superscripts (A–D) within the same row indicate significant differences among sampling days for the same treatment group, whereas different lowercase superscripts (a–c) within the same column indicate significant differences among treatment groups on the same sampling day (*p* < 0.05).

**Table 5:** Serum superoxide dismutase (SOD) activity (U/mL) in ewes during breeding and non-breeding seasons following melatonin treatment (Mean ± SEM).

Season	Group	Day 0	Day 7	Day 14	Day 30
Breeding	I	146.85 ± 1.26 ^B^	145.99 ± 0.99 ^Ba^	134.83 ± 2.74 ^Aa^	146.03 ± 1.45 ^Ba^
	II	148.21 ± 1.31 ^A^	216.79 ± 4.25 ^Cb^	194.22 ± 4.62 ^Bb^	213.06 ± 4.29 ^Cc^
	III	151.06 ± 1.26 ^A^	203.93 ± 11.31 ^Bb^	329.79 ± 4.29 ^Cd^	180.49 ± 3.03 ^Bb^
	IV	148.99 ± 1.04 ^A^	296.86 ± 19.54 ^Cc^	252.62 ± 3.79 ^Bc^	181.11 ± 5.01 ^Ab^
Non-breeding	I	126.21 ± 3.89	127.12 ± 2.65 ^a^	132.72 ± 7.09 ^a^	127.88 ± 3.24 ^a^
	II	118.39 ± 2.31 ^A^	213.39 ± 12.97 ^Cb^	211.82 ± 6.69 ^Cb^	162.99 ± 7.62 ^Bb^
	III	123.70 ± 2.79 ^A^	265.63 ± 12.84 ^Cc^	239.67 ± 5.37 ^Cc^	162.33 ± 6.61 ^Bb^
	IV	126.82 ± 3.03 ^A^	294.33 ± 13.82 ^Dc^	245.04 ± 5.18 ^Cc^	171.15 ± 11.02 ^Bb^

Within each season, different uppercase superscripts (A–D) within the same row indicate significant differences among sampling days for the same treatment group. Different lowercase superscripts (a–d) within the same column indicate significant differences among treatment groups on the same sampling day (*p* < 0.05). Cells without superscripts indicate that no significant differences were detected (*p* > 0.05).

**Table 6:** Serum catalase activity (U/mL) in ewes during breeding and non-breeding seasons following melatonin treatment (Mean ± SEM).

Season	Group	Day 0	Day 7	Day 14	Day 30
Breeding	I	16.42 ± 0.37 ^A^	15.89 ± 0.36 ^Aa^	16.82 ± 0.63 ^ABa^	18.42 ± 0.49 ^Ba^
	II	16.43 ± 0.34 ^A^	23.82 ± 0.59 ^Cb^	20.03 ± 0.64 ^Bb^	21.46 ± 0.63 ^Bb^
	III	16.81 ± 0.54 ^A^	24.79 ± 0.59 ^Cb^	26.26 ± 0.63 ^Cc^	21.75 ± 0.45 ^Bb^
	IV	17.31 ± 0.43 ^A^	25.29 ± 0.34 ^Cb^	29.11 ± 0.71 ^Dd^	20.95 ± 0.57 ^Bb^
Non-breeding	I	13.24 ± 0.43 ^A^	14.91 ± 0.42 ^a^	15.12 ± 0.41 ^a^	14.73 ± 0.59 ^a^
	II	12.96 ± 0.44 ^A^	18.65 ± 0.69 ^Bb^	20.64 ± 1.05 ^Bb^	18.46 ± 0.72 ^Bb^
	III	12.89 ± 0.51 ^A^	21.99 ± 0.55 ^Bc^	25.81 ± 0.84 ^Cc^	19.69 ± 0.46 ^Bbc^
	IV	13.09 ± 0.44 ^A^	22.72 ± 0.61 ^Bc^	27.55 ± 0.61 ^Cc^	21.47 ± 0.59 ^Bc^

Within each season, different uppercase superscripts (A–D) within the same row indicate significant differences among sampling days for the same treatment group. Different lowercase superscripts (a–d) within the same column indicate significant differences among treatment groups on the same sampling day (*p* < 0.05). Cells without superscripts indicate that no significant differences were detected (*p* > 0.05).

## Data Availability

The data supporting the reported results of this study are available from the corresponding author upon reasonable request.
